# Incidence and mortality of cutaneous melanoma stratified by sex, age, and age group – An analysis of the Brazilian population from 2013 to 2023^؆^

**DOI:** 10.1016/j.abd.2026.501338

**Published:** 2026-04-29

**Authors:** Débora Terra Cardial, Francisco Macedo Paschoal, Luiz Vinicius de Alcantara Sousa

**Affiliations:** a Department of Dermatology, Centro Universitário FMABC, Santo André, SP, Brazil; b Department of Collective Health, Centro Universitário FMABC, Santo André, SP, Brazil

**Keywords:** Brazil, Epidemiology, Incidence, Melanoma, Mortality

## Abstract

**Background:**

Cutaneous melanoma remains the leading cause of skin cancer-related mortality worldwide. Despite national awareness campaigns launched by the Brazilian Society of Dermatology since 1999, morbidity and mortality in Brazil remain substantially high.

**Methods:**

We conducted an ecological study using secondary data from DATASUS (Hospital Information System – SIH/SUS, Mortality Information System – SIM/SUS, and population estimates from IBGE). Melanoma cases were identified using ICD-10 code C43 between 2013 and 2023. Incidence rates were calculated per 100,000 inhabitants.

**Results:**

A total of 20,087 melanoma cases were reported during the study period. Men accounted for 57.5% of cases, a proportion that remained stable throughout the decade. Annual case counts increased from 1,547 in 2013 to 2,047 in 2023, representing a 32% rise. The South and Southeast regions — historically shaped by European immigration and concentrating the majority of White individuals — reported the highest incidence and mortality, consistent with prior population-based cohort data from southern Brazil.

**Conclusions:**

These findings highlight a multifactorial scenario in which demographic, behavioral, biological, and structural healthcare determinants converge to shape melanoma burden in Brazil. Sex-specific behavioral factors (lower photo protection adherence, delayed dermatological evaluation among men) and biological differences may contribute to male predominance. Expanding access to early-detection strategies and equitable resource allocation remains essential in a country marked by vast territorial, climatic, and socioeconomic heterogeneity.

Dear Editor,

Cutaneous melanoma accounts for nearly 90% of all skin cancer-related deaths worldwide.^1^ When detected at early stages, prognosis is significantly better, underscoring the importance of population-level screening. Globally, both incidence and mortality have shown a sustained upward trend, with the greatest burden concentrated in high-income regions.^2^ Among these, Australia remains a global reference due to the exceptionally high incidence of melanoma and the early implementation of nationwide screening and education campaigns, initiated in the 1980s. These efforts likely contributed to the observed peak in incidence in 2005, followed by a gradual decline.^3^ In contrast, other socioeconomically developed nations, including the United States, have not yet achieved similar reductions in incidence.^4^

In Brazil, the Brazilian Society of Dermatology launched national skin cancer awareness and early detection campaigns beginning in 1999. Despite these initiatives, morbidity and mortality related to melanoma remain substantially high.^5^ Genetic susceptibility, chronic ultraviolet exposure, and the country’s vast territorial and climatic heterogeneity all contribute to melanoma risk. Meanwhile, access to genetic risk testing and advanced diagnostic technologies remains limited across Brazilian territory, reflecting broader structural inequalities in the healthcare system.^6^

Given this scenario, understanding sociodemographic determinants of melanoma risk is essential to support targeted screening, equitable resource allocation, and the design of cost-effective public health strategies ‒ particularly in large and unequal countries such as Brazil. High-income nations, such as Australia, have already demonstrated that structured policies can lead to stabilization or reduction in melanoma mortality.^3^ This reinforces the urgency of implementing scalable and accessible early-detection strategies in Brazil.

Previous studies examining melanoma trends in Brazil have suggested that population growth and aging may be outpacing improvements in diagnostic capacity, potentially contributing to underdiagnosis or delayed detection in certain regions.^5^, ^7^, ^8^ To contribute to this discussion, we conducted an ecological study using secondary data from DATASUS, which is the public database of the Brazilian Ministry of Health. Case counts were extracted from the Hospital Information System (SIH/SUS), deaths from the Mortality Information System (SIM/SUS), and annual population estimates from the Brazilian Institute of Geography and Statistics (Instituto Brasileiro de Geografia e Estatística ‒ IBGE). Melanoma cases were identified using the ICD-10 code C43, and rates were calculated per 100,000 inhabitants.

Between 2013 and 2023, a total of 20,087 melanoma cases were reported in Brazil. A slight male predominance was observed, with men accounting for 57.5% of cases and women 42.5%. This sex distribution remained stable over the decade. Annual case counts increased gradually from 1,547 in 2013 to 2,047 in 2023 ‒ an overall increase of approximately 32% (Table 1).

**Table 1:** Melanoma cases per sex, 2013–2023*.

Sex	2013	2014	2015	2016	2017	2018	2019	2020	2021	2022	2023	Total
Male	903 (58.37)	916 (56.93)	1012 (56.41)	1036 (58.43)	1031 (56.19)	1038 (57.99)	1159 (58.59)	1120 (58.24)	1057 (57.70)	1102 (56.25)	1176 (57.45)	11,550 (57.5)
Female	644 (41.63)	693 (43.07)	782 (43.59)	737 (41.57)	804 (43.81)	752 (42.01)	819 (41.41)	803 (41.76)	775 (42.30)	857 (43.75)	871 (42.55)	8,537 (42.5)
Total	1547 (100)	1609 (100)	1794 (100)	1773 (100)	1835 (100)	1790 (100)	1978 (100)	1923 (100)	1832 (100)	1959 (100)	2047 (100)	20,087 (100)

* Values presented in Absolute and relative frequency.

Mortality rates also demonstrated consistent sex disparities. Age-adjusted mortality was higher among men throughout the study period, ranging from 0.93 to 1.14 deaths per 100,000 inhabitants, whereas female mortality ranged from 0.63 to 0.80. Although small year-to-year oscillations were observed, the persistent difference suggests a systematically elevated risk of melanoma-related death among men in Brazil (Table 2).

**Table 2:** Melanoma incidence per 100 000 inhabitants, by sex, from 2013 to 2023.

Sex	2013	2014	2015	2016	2017	2018	2019	2020	2021	2022	2023
Male	0.78	0.77	0.79	0.79	0.76	1.84	2.82	2.42	2.41	2.72	2.89
Female	0.60	0.59	0.58	0.57	0.55	1.75	2.83	2.37	2.33	2.73	3.01

Age was strongly associated with mortality. Individuals younger than 30-years accounted for fewer than 2% of deaths. Mortality increased markedly after age 50, with progressive escalation across older age groups. Adults aged 80-years and older represented the highest mortality burden, accounting for 22.9% of all deaths between 2013 and 2023. Additionally, the relative contribution of the ≥80-year-old group increased over time ‒ from 19.3% of deaths in 2013 to 24.9% in 2023 ‒ highlighting the growing influence of population aging on melanoma mortality in Brazil (Table 3, Table 4).

**Table 3:** Melanoma incidence per 100,000 inhabitants by age group, 2013–2023.

Age group (years)	2013	2014	2015	2016	2017	2018	2019	2020	2021	2022	2023
0–19	0.03	0.02	0.03	0.02	0.02	0.07	0.14	0.10	0.11	0.10	0.14
20–24	0.10	0.11	0.09	0.07	0.07	0.30	0.38	0.29	0.24	0.30	0.32
25–29	0.19	0.16	0.17	0.20	0.14	0.41	0.57	0.57	0.47	0.46	0.47
30–34	0.25	0.34	0.26	0.26	0.24	0.57	0.98	0.70	0.67	0.66	0.72
35–39	0.43	0.46	0.46	0.39	0.39	0.89	1.34	1.05	1.08	1.06	1.14
40–44	0.81	0.63	0.66	0.62	0.57	1.36	2.03	1.72	1.47	1.55	1.79
45–49	1.09	0.80	1.15	0.94	0.80	1.91	2.95	2.36	2.53	2.53	2.78
50–54	1.38	1.39	1.28	1.21	1.10	2.83	4.40	3.61	3.21	3.78	4.04
55–59	2.01	1.86	1.71	1.69	1.61	3.93	6.01	5.03	4.63	5.48	5.38
60–64	2.27	2.24	2.11	2.12	2.14	4.96	7.98	6.98	6.96	7.32	7.95
65–69	2.92	2.52	2.98	2.79	2.64	6.83	10.18	8.60	8.48	9.73	10.16
70–74	2.55	3.23	2.63	3.06	3.12	8.27	12.71	11.00	10.56	13.19	13.35
75–79	3.75	3.55	3.28	3.38	2.78	10.35	15.82	12.44	12.59	14.47	16.28
80 and older	1.93	2.47	2.33	2.17	2.79	10.45	16.23	13.58	13.98	16.55	17.08

**Table 4:** Melanoma mortality per 100,000 inhabitants by age group, 2013–2023.

Age group (years)	2013	2014	2015	2016	2017	2018	2019	2020	2021	2022	2023
0–19	0.01	0.01	0.02	0.02	0.01	0.01	0.01	0.01	0.02	0.01	0.01
20–24	0.06	0.07	0.04	0.09	0.07	0.04	0.05	0.07	0.04	0.04	0.06
25–29	0.11	0.12	0.12	0.13	0.12	0.09	0.10	0.10	0.10	0.12	0.10
30–34	0.27	0.22	0.23	0.25	0.23	0.19	0.21	0.16	0.19	0.23	0.20
35–39	0.41	0.40	0.40	0.42	0.35	0.36	0.28	0.31	0.31	0.31	0.31
40–44	0.51	0.63	0.61	0.59	0.51	0.49	0.48	0.43	0.40	0.43	0.40
45–49	0.72	0.87	0.91	0.81	0.84	0.80	0.79	0.69	0.70	0.86	0.75
50–54	1.07	0.98	1.29	1.45	1.21	1.15	1.13	1.07	0.93	1.02	1.10
55–59	1.72	1.63	1.76	1.61	1.51	1.37	1.54	1.64	1.53	1.42	1.40
60–64	2.37	2.23	2.49	2.23	2.38	2.18	2.42	2.09	2.08	2.07	2.32
65–69	3.10	3.39	3.26	2.87	3.05	2.80	3.37	2.96	2.75	2.66	2.83
70–74	4.12	3.74	4.87	3.89	3.93	4.04	4.57	4.24	3.73	4.58	4.14
75–79	5.42	5.30	5.52	6.08	5.66	6.02	5.58	6.17	4.86	5.30	5.54
80 and older	8.98	9.60	10.67	10.31	10.89	10.53	11.42	10.89	10.51	10.63	11.13

Ethnicity also played an important role. Self-declared White individuals consistently exhibited the highest mortality rates, representing 77%–81% of deaths across all years analyzed (Table 5).

**Table 5:** Melanoma deaths by ethnicity, 2013–2023.

Ethnicity	2013	2014	2015	2016	2017	2018	2019	2020	2021	2022	2023	Total
**White**	1,262 (81.58)	1,305 (81.11)	1,439 (80.65)	1,430 (80.65)	1,464 (79.78)	1,418 (79.17)	1,563 (79.02)	1,485 (77.22)	1,424 (77.73)	1,576 (80.45)	1,599 (78.11)	15,965 (79.48)
**Black**	38 (2.46)	34 (2.11)	42 (2.34)	41 (2.31)	50 (2.72)	32 (1.79)	62 (3.13)	47 (2.44)	42 (2.29)	45 (2.30)	49 (2.39)	482 (2.40)
**Asian**	8 (0.52)	2 (0.12)	5 (0.28)	1 (0.06)	7 (0.38)	3 (0.17)	4 (0.21)	7 (0.36)	5 (0.27)	6 (0.31)	7 (0.34)	55 (0.27)
**Brown**	172 (11.12)	189 (11.75)	243 (13.71)	243 (13.71)	257 (14.01)	267 (14.94)	307 (15.52)	303 (17.58)	316 (17.25)	307 (15.67)	359 (17.54)	3,030 (14.95)
**Indigenous**	1 (0.06)	2 (0.12)	1 (0.06)	0 (0)	4 (0.22)	1 (0.06)	2 (0.10)	3 (0.16)	0 (0)	1 (0.05)	1 (0.05)	15 (0.07)
**Unreported**	66 (4.27)	77 (4.79)	59 (3.29)	58 (3.27)	53 (2.89)	70 (3.91)	40 (2.02)	43 (2.24)	45 (2.46)	25 (1.28)	32 (1.56)	568 (2.83)
Total	1,547 **(100)**	1,609 **(100)**	1,794 **(100)**	1,773 **(100)**	1,835 **(100)**	1,791 **(100)**	1,978 **(100)**	1,923 **(100)**	1,832 **(100)**	1,959 **(100)**	2,047 **(100)**	**20,088 (100)**

Data are expressed as absolute numbers and percentages of melanoma-related deaths by ethnicity between 2013 and 2023.

Brazil’s heterogeneous ethnic distribution helps contextualize this pattern. The South and Southeast regions ‒ historically shaped by extensive European immigration ‒ concentrate the majority of White individuals in the country and consequently report the highest melanoma incidence and mortality.^9^ This demographic pattern aligns with findings from our national dataset and helps explain regional differences in melanoma burden.

Our findings are consistent with the long-standing epidemiological profile described in a large population-based cohort from Blumenau, Santa Catarina, which followed melanoma incidence over nearly four decades. That study demonstrated a persistent predominance of melanoma among White men older than 54-years, with incidence increasing steadily throughout the observation period.^9^ The similarity between our national findings and the Blumenau cohort reinforces the hypothesis that demographic composition ‒ particularly the high concentration of individuals of European descent in southern Brazil ‒ plays a substantial role in shaping the distribution of melanoma in the country.

In addition to environmental and demographic factors, sex-specific behavioral and biological determinants may contribute to the disproportionate burden among men. International studies have shown that men tend to engage less frequently in photoprotection, delay dermatological evaluation, and have lower adherence to preventive measures.^10^ Biological susceptibility has also been proposed: differences in immune response, DNA repair capacity, and UV-radiation-induced carcinogenesis may predispose men to more aggressive tumor behavior, regardless of geographic or socioeconomic context.^9^, ^10^ Together, these factors may help explain the higher mortality observed among Brazilian men.

Taken as a whole, our findings illustrate a multifactorial scenario in which demographic, behavioral, biological, and structural healthcare determinants converge to shape melanoma burden in Brazil. If current global patterns are maintained, Brazil may follow the trajectory of high-income countries, with potential stabilization or gradual decline in incidence among younger individuals, but a continued rise in cases among adults older than 55-years due to population aging. With improved public awareness and expanded screening strategies, however, a reduction in mortality is possible, as demonstrated in other national experiences.^1^, ^2^, ^3^

## ORCID IDs

Francisco Macedo Paschoal: 0000-0002-6264-1538

Luiz Vinicius de Alcantara Sousa: 0000-0002-6895-4914

## Financial support

This study was financed in part by the Coordenação de Aperfeiçoamento de Pessoal de Nível Superior - Brazil (CAPES) - Finance code 001 - and by the researchers' own resources.

## Authors' contributions

Débora Terra Cardial: Conception and study design; data collection, analysis, and interpretation; statistical analysis; manuscript drafting or critical revision for important intellectual content; data acquisition, analysis, and interpretation; active participation in research supervision; intellectual contribution to the diagnostic and/or therapeutic management of the studied cases; critical literature review; final approval of the submitted version of the manuscript.

Francisco Macedo Paschoal: Conception and study design; active participation in research supervision; intellectual contribution to the diagnostic and/or therapeutic management of the studied cases; final approval of the submitted version of the manuscript.

Luiz Vinicius de Alcantara Sousa: Conception and study design; data collection, analysis, and interpretation; statistical analysis; data acquisition, analysis, and interpretation; active participation in research supervision; critical literature review; final approval of the submitted version of the manuscript.

## Research data availability

The entire dataset supporting the results of this study was published in this article.

## Conflicts of interest

None declared.
